# Supplementary material for: Patterns of Intron Gain and Loss in Fungi
Source: PLoS Biol. 2004 Nov 30;2(12):e422. doi: 10.1371/journal.pbio.0020422 (PMC532390; doi:10.1371/journal.pbio.0020422)
Supplement: Table S1 — Also available at http://genes.mit.edu/NielsenEtAl/. (4.3 MB ZIP). [file pbio.0020422.st001.zip › NielsenEtAl/html/189.html]

AN2272.1.NCU00414.1.MG06270.1.FG06932.1


```
 CLUSTAL W (1.82) Multiple Sequence Alignments - Introns Inserted


Sequence 1: MG06270.1	351 aa
Sequence 2: FG06932.1	428 aa
Sequence 3: NCU00414.1	479 aa
Sequence 4: AN2272.1	352 aa
Alignment Length: 485 aa
Number Identitical Residues: 195 aa
Alignment Score (without introns) 9449


MG06270.1 	------------------------------------------------------------
NCU00414.1	MLLTHPITRPQRLIAGKLESLIGVSYAVGARRHGQPSAAGATRCFYSTAPVNPFPAANPS
FG06932.1 	MMLTKPSSSL------KLPSLT-------------PTYRTWTRSFASVTPVR-LPKKQAP
AN2272.1  	------------------------------------------------------------
          	                                                            

MG06270.1 	----------------------------------------------MSSPKEFRLFCLEN
NCU00414.1	TSNLVRGCDPAVGCFSLSASNLIPVNNSFHTTTPSRFSIRSNPFSKMAATKDYRLLCLEN
FG06932.1 	RLNPTGAVDPSI-RLKLFPSKATPL---FVSTSPYRV-YQTRQLSTMSSVKEYSLFCLEN
AN2272.1  	----------------------------------------------MAAPQGYPLLCLEN
          	                                              *:: : : *:****

MG06270.1 	PLLD1IQAVG~D-------------------------E~ALLNKYGLKANDAILAEAKHA
NCU00414.1	PLLV1RAHPG~FPYSQSASQHHGATLRPPSFARTCMMQ0ALLEKYGLKANDAILAEEKHQ
FG06932.1 	PLLD1IQAQG~D-------------------------Q~ALLDKYGLKPNDAILAEEKHI
AN2272.1  	PLLD1IQAVG2D-------------------------D~SLLEKYGLKANDAILAEEKHM
          	***      *                           : :**:*****.******* ** 

MG06270.1 	GIFEELLNNYDAKLIPGGAAQNTARGAQ~YMLPPNSVVYSGGVGDDKYSAILRDAVQKVG
NCU00414.1	GLFEDLLQNYDAKLIAGGAAQNTARGAQ~YLLPPNSVVYLGGVGDDKYAAILHDAVKQAG
FG06932.1 	PLYEDLLNNYDAKLIAGGAAQNSARGAQ~YILPPNSVVYVGGAGDDKYSAILHDAVKAAG
AN2272.1  	GLYEELLQHRDAKLIAGGAAQNTARGAQ0YILPDNSTLYIGCVGKDKYADILQDACKKAG
          	 ::*:**:: *****.******:***** *:** **.:* * .*.***: **:** : .*

MG06270.1 	LRVEYRVDPKETTGRCAVVITGHNRSMCTELGAANHYEVEFLKKPEVWELVKGAEVYYIG
NCU00414.1	LRVEYRVDPKISTGRCGVVITGHNRSMCTELGAANHYDLEHLKKPEVWSLVENAEVYYVG
FG06932.1 	LRVEYRVDPKEKTGRCGAIITGHNRSLCTDLGAANHYDLDHLKKPEIWKLVENAEVYYVG
AN2272.1  	VHTEYRVDDAQPTGKCGVIITGHNRSMCTHLAAANEYKVDHLKQPHIWSLVEKAQYYYVG
          	::.*****    **:*..:*******:**.*.***.*.::.**:*.:*.**: *: **:*

MG06270.1 	GYHFTV1CPPAIMELAKEAATNNK~TFILSLSAPFIPQFFKDPLDAALPYCDYVIGNETE
NCU00414.1	GYHFTV~CPPAIMELAKQAASGNK~PFILSLSAPFICQFFKEPLDASAPYWDYVIGNEGE
FG06932.1 	GFHFTV1CPPAIMELAKQAAKDNK~PFVLSLSAPFIPQFFKEVVDASAPFWDYIIGNETE
AN2272.1  	GYHLTV~CVPAIQALGEEAAAKNK0VFMLSLSAPFIPQFFKDQLDSVLPYTDYTFCNETE
          	*:*:** * ***  *.::**  **  *:******** ****: :*:  *: ** : ** *

MG06270.1 	AAAFAESHDMADKKDDVRAIAKAIANLPKENSQRKRVAIITQGTLPTIVAVQ---GEEEV
NCU00414.1	AAAYAESHGLNTT--DVKEIAKALANLPKENTQRKRVAIITQGTEPTIVAIQ---GEDEV
FG06932.1 	AAAYAESHDLPSK--EPKDVAKHLANLPKENSQRKRVAIITQGTDPTLVAIQ---GEDEI
AN2272.1  	AVAYAESHEWGTT--DIVEIAKKLAQLPKKNTNRSRIAVVTQGTLPTITATVTTSGEVEV
          	*.*:****    .  :   :** :*:***:*::*.*:*::**** **:.*  :::** *:

MG06270.1 	KEYPVHEIAKEQINDTNGAG~DAFAAGFCAGVVENKALAECVDRGQWLARLSIQELGPS2
NCU00414.1	KEYPVHSIDPAKINDTNGAG~DAFAGGFAAGVVEGKSIEESIHMGQWLAKLSIQELGPS2
FG06932.1 	KEFPVHAIETEKINDTNGAG~DAFAGGLLAGILQGKPLETSIDMGQWLARLSIQELGPS2
AN2272.1  	KEFPVHEISKDAINDTNGAG2DAFAGGFVAGVVQGKSLEESVDLGQWLAKLSIQELGPS2
          	**:*** *    ******** ****.*: **:::.*.:  .:. *****:********* 

MG06270.1 	YPFPKQTYQPKSA
NCU00414.1	YPFPKQAYPGHN-
FG06932.1 	YPFPKQTYQAA--
AN2272.1  	YPFPKQTYTSGRS
          	******:*    :
```
